# Item

**Published:** 1901-11

**Authors:** 


### ITEM.

The Rio Chemical Company announces the removal of their office from St. Louis to 56 Thomas Street, New York City. The reasons for this change as given by this celebrated house are that their business has grown to such proportions, that they desire to be where they could have better facilities for procuring the various ingredients that enter into the composition of their preparations and thus preserve that uniformity that is so important, and to be more in touch with their foreign offices. Their foreign business has grown to such proportions, that there is scarcely a country in the world where educated physicians do not use their products.

